# The search for an elusive worm in the tropics, the past as a key to the present, and reverse uniformitarianism

**DOI:** 10.1038/s41598-019-54643-8

**Published:** 2019-12-05

**Authors:** Luis I. Quiroz, Luis A. Buatois, Koji Seike, M. Gabriela Mángano, Carlos Jaramillo, Andrew J. Sellers

**Affiliations:** 1 Department of Geological Sciences, University of Saskatchewan, Saskatoon, SK S7N 5E2 Canada; 2 Geological Survey of Japan, National Institute of Advanced Industrial Science and Technology (AIST), Central 7, 1-1-1 Higashi, Tsukuba, Ibaraki 305-8567 Japan; 3 Smithsonian Tropical Research Institute, Panama City, Panama; 4 ISEM, U. Montpellier, CNRS, EPHE, IRD, Montpellier, France; 5 Department of Biology, McGill University, Montreal, Québec, Canada; 6 Present Address: Ecopetrol, Cra. 7 # 32-42, Bogotá, Colombia

**Keywords:** Evolutionary ecology, Solid Earth sciences

## Abstract

The distribution of trace-making organisms in coastal settings is largely controlled by changes in physicochemical parameters, which in turn are a response to different climatic and oceanographic conditions. The trace fossil *Macaronichnus* and its modern producers are typical of high-energy, siliciclastic foreshore sands in intermediate- to high-latitude settings characterized by cold-water conditions. However, it has been found in Miocene Caribbean deposits of Venezuela, prompting the hypothesis that upwelling of cold, nutrient-rich waters rather than latitude was the main control of its distribution. To test this hypothesis that was solely based on the fossil record, several trenches and sediment peels were made in two high-energy sand beaches having different oceanographic conditions along the Pacific and Caribbean coasts of the Central American Isthmus. As predicted, the burrows were found only in the highly productive waters of the Pacific coast of Costa Rica in connection with upwelling, while they were absent from the warm, oligotrophic waters of the Caribbean coast of Panama. This finding demonstrates that sometimes the past may be a key to the present, providing one of the few documented examples of reverse uniformitarianism.

## Introduction

Trace fossils record organism behavior as a response to the dynamics of physical and chemical conditions during deposition, thus providing a wealth of information to reconstruct the ecology and environments of the past^1,2^. Although trace fossils in coastal environments are highly indicative of ecology and environment, shallow-marine bioturbation structures have not been adequately evaluated as paleoclimatic indicators. However, Goldring *et al*.^3^ explored the present distribution of burrowing crustaceans and infaunal echinoids in nearshore settings, establishing latitudinal limits for their well-known biogenic structures, with *Ophiomorpha* generally restricted to the tropical and subtropical zones, *Thalassinoides* and *Scolicia* extending into temperate areas, and the arctic regions having only mollusk and polychaete traces. Other lines of evidence have suggested an increase in trace-fossil size towards high latitudes and more common equilibrichnia in temperate settings^4,5^. Furthermore, the peculiarities of bioturbation in marine settings affected by glacial processes have been reviewed in detail^6^. In comparison, the study of latitudinal patterns in the distribution of bioerosion structures has a longer tradition because the analysis of drill holes in shells provides evidence of predation pressure, which has been historically regarded to be higher in the tropics and declining towards the poles^7^, although a more nuanced view has emerged subsequently^8–10^. More recently, trace fossils have been used as paleoclimatic indicators as a line of evidence in a multidisciplinary study of a Pleistocene shallow-marine succession^11^.

The ichnogenus *Macaronichnus* comprises small, cylindrical, unbranched burrows, straight, sinuous or very rarely spiral, and typically oriented parallel to stratification. It is actively filled by light-colored sand, which contrasts with the dark surrounding mantle. *Macaronichnus* is produced by the deposit-feeding activities of opheliid polychaetes. Both biogenic structures and their modern producers are characteristic of intermediate- to high-latitude foreshore settings^4,5,12,13^. However, Quiroz *et al*.^12^ recorded an unusual occurrence of this ichnogenus in Miocene tropical deposits of Venezuela, representing a departure from the currently accepted model. These authors linked this occurrence to upwelling of cold, nutrient-rich waters and the high primary productivity of the Caribbean Sea before the final uplift of the Panama Isthmus. In addition, they predicted the presence of *Macaronichnus* along modern high-energy, siliciclastic shorelines in the tropical region subject to similar oceanographic conditions as interpreted from the sedimentary record.

Based on previous work^12^, we formulate null and alternative hypotheses. The null hypothesis postulates that *Macaronichnus* is found across all high-energy beaches in Central America. The alternative hypothesis posits that *Macaronichnus* occurs only on the Pacific coast and not on the Caribbean coast. In this study, we tested these hypotheses by searching for these polychaete traces in two high-energy beaches of the Caribbean coast of Panama and the Pacific coast of Costa Rica. The contrasting oceanographic conditions of these tropical coasts offer a unique opportunity to support the proposed oceanographic value of this ichnogenus. Uniformitarianism, the principle stating that the present is the key to the past, represents a milestone in geology and palaeontology^14,15^. The opposite situation, using the fossil record to state a prediction for modern environments, is rare^16^, and here we present such a case.

## Oceanographic Context

**Figure 1:**
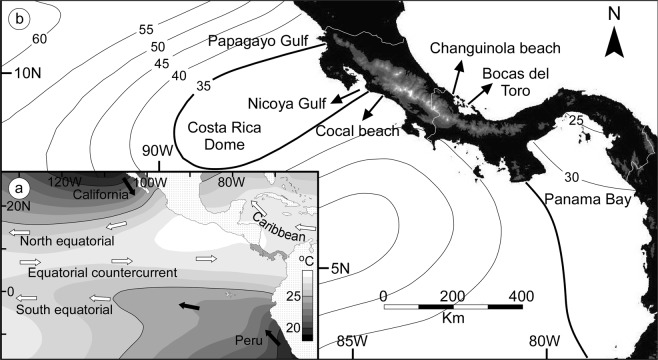
Hydrographic context and location of study area. **(a)** Map of mean annual sea surface temperature and major oceanic currents in tropical America (modified from^19^). **(b)** Location map of the study sites in Panama and Costa Rica showing areas with relief above 500 m in gray shade and above 3000 m in white. Contour lines depict the average thermocline depth for March (20 °C isotherm) in the region of the Costa Rica Dome according to Fiedler^24^. Relief map in Fig. 1b made with GeoMapApp (www.geomapapp.org)/CC BY/CC BY^63^.

The tropical oceans along the Central American Isthmus (CAI) display markedly different oceanographic conditions, which are reflected in shallow-marine communities with contrasting ecological structure^17^. Despite intense rainfall (>3000 mm year^−1^) mostly derived from the Intertropical Convergence Zone (ITCZ) over Panama and Costa Rica from May to December^18,19^, abundant nutrients supplied by freshwater runoff are mainly found in areas protected from open ocean influence^18,20^. Although both areas experience high mean annual sea surface temperatures (Fig. 1a), the basin-scale thermocline shoaling and local wind-driven upwelling on the Pacific side^19^ result in a high supply of nutrients in the euphotic zone, thus elevating primary production in shallow waters^21,22^. During the dry season from January to April (boreal winter), when the ITCZ migrates southward and northeasterly trade winds blow through low passages across the CAI adjacent to the Gulfs of Papagayo and Nicoya, and the Panama Bay (Fig. 1b), the thermocline nearly breaks at the surface, producing strong upwelling of nutrient-rich cold waters, resulting in phytoplankton blooms in surface waters^21–23^. Additionally, there is a permanent thermocline ridge off the coast of Costa Rica known as the Costa Rica Dome (Fig. 1b), which shoals gradually toward the coast during this season^24^. Along the coast, between the Gulf of Nicoya and the Panama Bay, upwelling is hampered by higher mountain ranges that effectively block the trade winds. However, nutrient-rich thermocline water does rise to subsurface depths (up to 25 m) during the dry season, leading to phytoplankton blooms at these levels^22^. Increase in nutrients and high planktonic productivity favors the growth of seaweeds and plankton-feeding organisms over coral reefs and seagrasses^17^.

The Caribbean Sea, on the contrary, is characterized by nutrient-poor, clear waters throughout the year, and is considered in general as an oligotrophic sea^18^. The warm Caribbean current mixes the surface water (Fig. 1a), resulting in a mass of warm (>20 °C) and salty water in the upper 200 m throughout the year^25^. Accordingly, extensive coral reefs and seagrass beds dominate benthic ecosystems, although high freshwater and sediment discharge by rivers inhibit their development in nearshore areas^17,18^.

## Occurrence of *Macaronichnus*: Sedimentologic and Hydrodynamic Setting

**Figure 2:**
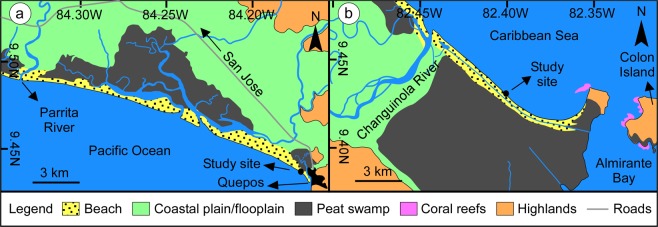
Detailed map with sedimentary environments and location of study sites. **(a)** Cocal Beach, Costa Rica. Map Data: Google, 2016 CNES/Astrium. **(b)** Changuinola Beach, Panama. Map Data: Google, 2016 CNES/Astrium, 2016 DigitalGlobe. Extent of Changuinola swamp from^26^.

Two high-energy beaches exposed to open ocean swells, but under contrasting upwelling conditions, were examined (Table 1). Sands in both areas are compositionally immature, consisting entirely of siliciclastic sediment with approximately 60–70% mafic grains and 30–40% felsic grains. Changuinola Beach, on the Caribbean coast of Panama, is not affected by upwelling. This beach lies immediately to the west of the Bocas del Toro Archipelago (Fig. 2b) and forms a prograding, wave-dominated strandplain system^26^. The area experiences a microtidal range (<0.5 m), with a well-developed surf zone and moderate- to high-energy waves (0.5–1 m). The study site is located 6 km southeast of the Changuinola River (9.43°N/82.40°W), with a foreshore characterized by moderately to well-sorted, fine- to medium-grained, subangular sands, corresponding to an intermediate morphodynamic state^27^. Despite appropriate hydrodynamic conditions, biogenic structures attributable to *Macaronichnus* are absent.

**Table 1:** Comparison of environmental parameters in the two sites studied.

Environmental Parameters	Changuinola Beach (Caribbean)	Cocal Beach (Pacific)
Wave energy	well-developed surf zone; moderate- to high-energy waves (0.5–1 m)	moderately developed surf zone; moderate- to high-energy waves (0.5–2 m)
Tidal range	microtidal (<0.5 m)	mesotidal (<3.0 m)
Proximity to a river mouth	6 km southeast of the Changuinola River	in front of small, estuarine mangrove systems and river mouths
Upwelling	unaffected	affected
Sediment composition	siliciclastic (immature sand)	siliciclastic (immature sand)
Climate	humid-tropical; temperature averages 26.0 °C, with a ± 3° range; annual precipitation 3000 mm	humid-tropical; temperature averages 26.7 °C, with a ± 3° range; annual precipitation 3700 mm

**Figure 3:**
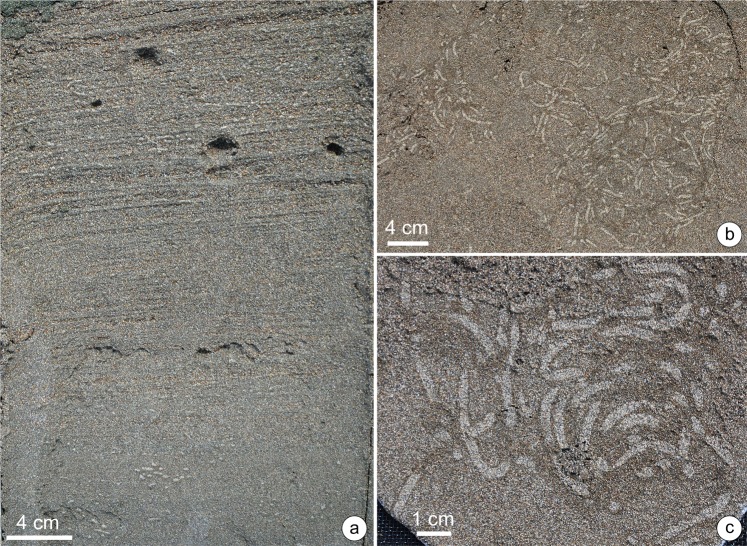
Incipient *Macaronichnus* from Cocal Beach. **(a)** Box core of foreshore sediments displaying low-angle cross-stratification and planar lamination, with localized zones of intense bioturbation by *Macaronichnus*. **(b)** Horizontal view of the *Macaronichnus* level. **(c)** Detail of resin peels showing the typical light-colored sand trail contrasting with the dark surrounding mantle.

Cocal Beach, on the Pacific coast of Costa Rica, is affected by upwelling. This coast forms a series of transgressive barrier islands and spits in front of small, estuarine mangrove systems and river mouths^28^, which extend for about 40 km to the rocky headlands of the Gulf of Nicoya in the west (Fig. 2a). A semidiurnal, mesotidal range (<3 m) and moderate- to high-energy waves (0.5–2 m) are typical in this area^28^; however, the surf zone tends to be narrower than at Changuinola beach. The study site is located to the west of Quepos (9.44°N/84.17°W), with a foreshore of up to 70 m wide during low tide, consisting of moderately sorted, medium-grained, subangular sands, characteristic of an intermediate morphodynamic state. Feeding traces assigned to *Macaronichnus* were found in this beach. The sediments with *Macaronichnus* occur at the mid foreshore level, 40 to 80 cm below the surface (Fig. 3), and display the typical, pervasive bioturbation observed in the sand beaches of temperate latitudes^1,29^. Burrow densities of up to 3600 burrows m^−2^ on a horizontally stripped section were recorded.

Although the influence of upwelling is not the only difference between the environmental conditions of the two sites, the other contrasting factor (i.e. tidal range) is not considered to have played a major role in controlling bioturbation by the *Macaronichnus* producer. This ichnogenus has been documented in coastal settings encompassing a wide variety of tidal ranges, from microtidal to mesotidal^29–31^. With respect to wave energy, both areas are affected by moderate- to high-energy waves and the slight difference between the two beaches in terms of size of waves is judged to be negligible based on overall occurrences of similar biogenic structures elsewhere.

## Deposit-feeding Polychaetes in Sand Beaches

The ichnogenus *Macaronichnus* is attributed to the deposit-feeding activities of opheliid polychaetes occurring in clean, intertidal sands, subject to moderate- to high-energy waves. Although these polychaetes are well known for having narrow preferences for particle size^32^, they occur in a wide variety of marine environments and depths^33^. However, only a few species of genera *Ophelia* and *Thoracophelia* (formerly *Euzonus*) live in dense populations in high-energy intertidal habitats and have been observed to produce *Macaronichnus*. In particular, *Thoracophelia furcifera* occurs on the Pacific coast of Costa Rica and has been detected in the Quepos area^34^.

Although *Ophelia limacina* lives in moderate numbers (180 individuals m^−2^) in sands of the outer estuarine channel at Willapa Bay, Washington^30^, other species including *Thoracophelia mucronota* in the Pacific coast of North America^35^, *Thoracophelia* sp. in Japan^29^ and *T. furcifera* in southern Brazil^36^ live in populations one to two orders of magnitude larger along sand beaches experiencing different morphodynamic states. Recorded densities at Cocal Beach are consistent with these latter values. In the fossil record, cross-bedded, outer estuarine channel or subtidal sandstone is sparsely bioturbated with *Macaronichnus* and typically accompanied by more diverse trace-fossil assemblages^37–39^, whereas ichnofabrics in the foreshore are commonly monospecific and display pervasive bioturbation^12,40^. Additionally, a distinction has been made between a small *Macaronichnus* (2–5 mm in diameter) characteristic of the foreshore, and a larger one (5–15 mm in diameter) more common in subtidal settings, which is produced by *Travisia japonica*, a common large travisiid polychaete of the northern Pacific Ocean^41^.

The bloodworm *T. mucronata* is by far the most abundant opheliid polychaete living in intertidal sands and holds a world record in population density, with thriving colonies of up to 43,000 individuals m^−2^ in California^35^. They are also the fastest burrowing polychaetes of their kind, with a gut throughput time of 4 minutes, which is an order of magnitude higher than that of other deposit-feeders of similar weight^42^. It appears that these worms ingest sand and feed from organic matter and microorganisms adhering to the grains, although diatoms are not included in their diet^35^. Interestingly, these polychaetes are able to meet their nutritional requirements by direct assimilation of detrital organic carbon derived from seaweeds, even in the absence of microbial modification^43^. This is unusual for deposit-feeders, which have long been thought to rely on microbial carbon obtained from bacteria rather than the refractory components of organic matter^44^. Although vascular plant detritus is very refractory, some components of seaweed detritus and pellets are enriched in nitrogen and are directly available to detritivores^45^. Despite the low organic content of high-energy sand beaches, these marine polychaetes inhabit the intertidal region where sand filtration of particulate organic material from the wave swash is greatest^27^.

## Discussion

Sandy beaches are extremely dynamic environments and are considered to be physically controlled ecosystems, providing habitats to a variety of organisms^27^. Although species richness, abundance and biomass of macrobenthic communities tend to increase from the harsh reflective beaches to the more benign dissipative ones^46^, *Macaronichnus* and their producers are present on the Hasaki coast of Japan even during times of reflective beach morphodynamic state, but displaying a more restricted distribution^29^. However, oceanographic variables such as temperature and primary production have not been integrated in deciphering latitudinal trends^46^. Temperate latitudes experience planktonic blooms during the spring season when stratification sets in and sunlight in the euphotic zone becomes sufficient, after the winter mixing of nutrients in surficial waters^21^. In California, which is one of the four major upwelling regions along the eastern boundaries of the ocean, these blooms are maintained during the summer due to the extended upwelling season^47^. This may explain the extreme occurrence of *T. mucronata* in this area.

Because temperature covaries with the amount of nutrients in upwelling areas, discriminating between these two parameters as controlling factors on bioturbation is not straightforward (Supplementary Fig. S1). Temperature measurements through the year in Pacific areas of Central America affected by upwelling show that the intertidal zone experiences both a drop of ~11 °C in water temperature and a marked increase in nutrient levels during the months of strong upwelling (Supplementary Fig. S1). Increase of nutrients supplied by the Changinola River in Bocas del Toro during the rainy season does not result in the occurrence of *Macaronichnus*, underscoring the role of upwelling as a controlling factor on bioturbation. The occurrence of *Macaronichnus* in the highly productive waters of the Pacific coast of Costa Rica, further supports this hypothesis and demonstrates that abundant nutrients and high primary productivity are important factors controlling the occurrence of deposit-feeding opheliid polychaetes in high-energy intertidal sands, an environment commonly dominated by filter-feeders and scavengers^27^. Additionally, *Macaronichnus* has been recently reported in foreshore and shallow-marine deposits from several units in two tropical localities, including Miocene and Pleistocene units in the Cape Verde Archipelago^48^ and Pliocene units in the Azores Archipelago^49^. These tropical localities are under the influence of the Canary Current upwelling system^50^, which is believed to have been active since at least the early Miocene, albeit with variable intensities^51^, further supporting the original suggestion^12^.

Although the ecological structure of tropical sand beaches has not been extensively studied, a few references on the polychaete fauna along the coasts of tropical America document the occasional occurrence of *T. furcifera* in one beach of the Pacific coast of Costa Rica and Colombia^34^. Other studies reported the occurrence of *T. mucronata* in Ecuador^52^ and *Thoracophelia* sp. in Peru^53^. These species are well known from the Pacific coast of North America and southern Chile^35,54^, where the cold and highly productive California and Peru currents, respectively, provide migratory pathways for these temperate polychaetes in the tropical Pacific Ocean.

The outcome of this study parallels the finding of kelp forests refugia in tropical, upwelled waters of the Galapagos Island, based on a sophisticated, synthetic oceanographic and ecophysiological model that predicted the occurrence of these high-latitude ecosystems in areas where bathymetry and upwelling resulted in thermocline shoaling^55^. Notably, the Pacific coast of Central America was among the predicted areas, whereas the oligotrophic waters of the Caribbean Sea were consistently excluded.

The potential of reverse uniformitarianism in ichnology has been barely underscored at a large scale^16^. The fact that certain biogenic structures are better expressed in the fossil record than in the modern (e.g. cumulative structures) is well known^56^. However, only in rare instances have ichnologists been able to put forward predictions based on the fossil record that have been subsequently favorably tested with modern data. A classic example is Seilacher’s^57^ suggestion that deep-sea graphoglyptids were pre-turbidite trace fossils formed as shallow-tier open burrows in the hemipelagic mud and ultimately preserved due to erosive removal of the uppermost muddy layer by a turbidity current and later casting with sand. At the time, this interpretation was entirely based on the fossil record. Later studies of the modern seafloor provided photographs of some of these structures^58,59^, but it was not until recently that the infaunal nature of these biogenic structures was confirmed based on submarine exploration of the Mid-Atlantic Ridge^60^. The alternative hypothesis for the occurrence of *Macaronichnus* has been refined after observations of its distribution in the fossil record and modern environments. However, the oceanographic conditions of the Caribbean region in the Miocene led to further predict its occurrence in tropical waters affected by upwelling^12^. Accordingly, the fossil record has yielded essential information for predicting the modern occurrence of *Macaronichnus* in the tropics, providing another example of reverse uniformitarianism, using the past as a key to the present.

## Conclusions

In this study, we document the occurrence of *Macaronichnus* in a high-energy sand beach in the highly productive waters of the Pacific coast of Costa Rica in connection with upwelling, and its remarkable absence from the oligotrophic waters of the Caribbean Sea. This finding challenges the conventional model of macrofaunal structure in exposed sand beaches as being exclusively controlled by the physical dynamics of these ecosystems, and instead prompts us to invoke upwelling and primary productivity as a major control for the occurrence of marine deposit feeders in an atypical environment. This study highlights the use of ichnology to infer ancient climatic conditions, showing that local oceanographic variables may play an important role at controlling the distribution of *Macaronichnus* than just the water temperature changes associated with latitude. We successfully tested a prediction based on the fossil record, demonstrating that, at least in some cases, the past may be indeed a key to the present, providing an example of reverse uniformitarianism.

## Materials and Methods

Two high-energy beaches exposed to open ocean swells, one affected by upwelling (Pacific Costa Rica) while the other one is not (Caribbean Panama), were chosen as study sites. Fieldwork was carried out during May 2010 in Costa Rica, and December 2010 in Panama. Digging of trenches, sediment cores and resin peels follow the methodology employed by Seike^29^ on the Pacific coast of Japan. To observe modern *Macaronichnus*, resin peels were prepared from vertical sections of sediment obtained using a specially designed boxcorer (24 cm wide, 60 cm deep, 10 cm thick). Resin peels enable detailed observations of physical and biogenic sedimentary structures, as they emphasize textural differences^29^. This procedure also yields undisturbed sediment samples from both intertidal and subtidal water-saturated sediments. We also dug trenches on the beach to make horizontal sediment peels of the *Macaronichnus*-bearing horizon.

Data on physicochemical conditions were measured in the field or obtained from the available literature^26,28^. Sea surface temperature (SST) data and water samples for nutrient analysis were obtained from an intertidal rocky shore in Culebra Point, Gulf of Panama (8° 54.71′N, 79° 31.76′W) a region known to be influenced by seasonal upwelling activity^22^. Temperatures were recorded every hour from December 2016 to June 2018 using Thermochron i-Button® dataloggers (Maxim Integrated Products, Sunnyvale, California, USA), which were replaced every four to six weeks. The dataloggers were waterproofed by coating them with a plastic cover. This method of waterproofing has a negligible effect on the dataloggers’ ability to accurately record temperature^61^. Once waterproofed, the dataloggers were attached to a rock face with marine epoxy at approximately one meter below the mean low-water height.

We collected two replicate water samples every two weeks in Culebra Point during the upwelling season of 2017 and 2018 (January-April), and during the non-upwelling season of 2016 and 2017 (September-December) from a depth of approximately one meter using a 250 ml acid-washed polyethylene bottle. The samples were filtered through 25-mm combusted Whatman glass-fiber filters with a pore size of 0.7 μm. We then froze the filtrate for later quantification of nitrate on a Lachat autoanalyzer^62^.

## Supplementary information

Supplementary Figure S1

## Data Availability

The datasets generated during the current study are available from the corresponding author on reasonable request.
