# Supplementary material for: The search for an elusive worm in the tropics, the past as a key to the present, and reverse uniformitarianism
Source: Sci Rep. 2019 Dec 5;9:18402. doi: 10.1038/s41598-019-54643-8 (PMC6895032; doi:10.1038/s41598-019-54643-8)
Supplement: Supplementary file 1 — Supplementary Figure S1 [file 41598_2019_54643_MOESM1_ESM.pdf]

The search for an elusive worm in the tropics, the past as a key to the present, and reverse uniformitarianism

Luis I. Quiroz[1]*, Luis A. Buatois[1], Koji Seike[2], M. Gabriela Mángano[1], Carlos Jaramillo[3,4], Andrew J. Sellers[3,5]

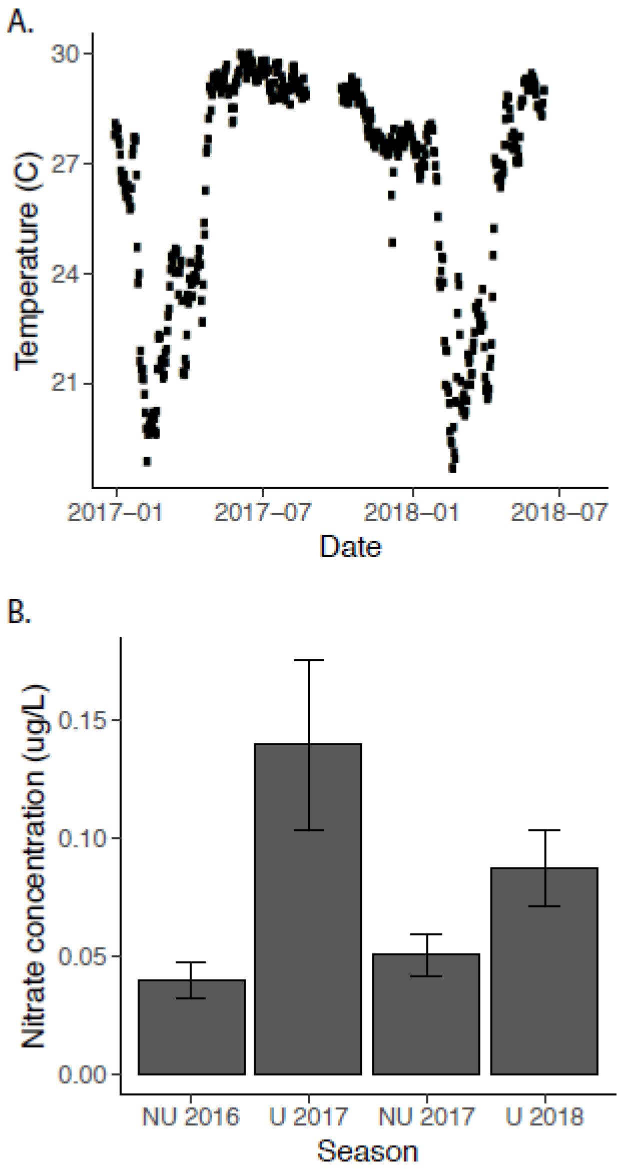

Figure S1. (**a**). Daily sea surface temperatures recorded in an intertidal rocky shore in the Gulf of Panama. Each point represents a daily mean calculated from hourly measurements. The figure shows seasonal cooling events generated by upwelling activity in that region. Temperatures were recorded at one meter below the mean low tide height. (**b**) Seasonal comparison of nitrate concentration (µg/L) in surface water in an intertidal rocky shore in the Gulf of Panama. Water samples were collected at a depth of approximately one meter during the non-upwelling seasons of 2016 and 2017 (NU 2016 and NU 2017, respectively), and during the upwelling season of 2017 and 2018 (U 2017 and U 2018, respectively). Vertical lines above bars represent standard errors.
